# Correction: Shi et al. Effect of Final Rolling Temperature on Microstructures and Mechanical Properties of AZ31 Alloy Sheets Prepared by Equal Channel Angular Rolling and Continuous Bending. *Materials* 2020, *13*, 3346

**DOI:** 10.3390/ma16155493

**Published:** 2023-08-07

**Authors:** Laixin Shi, Lei Liu, Li Hu, Tao Zhou, Mingbo Yang, Yong Lian, Jin Zhang

**Affiliations:** 1 College of Material Science and Engineering, Chongqing University of Technology, Chongqing 400054, China; shilaixin2016@cqut.edu.cn (L.S.); liulei@2017.cqut.edu.cn (L.L.); yangmingbo@cqut.edu.cn (M.Y.); 2 Institute for Advanced Materials and Technology, University of Science and Technology Beijing, Beijing 100083, China; liany09@126.com (Y.L.); zhangjin@ustb.edu.cn (J.Z.)

## Error in Figure

In the original publication [1], there was a mistake in Figure 3 as published. Because of the authors’ negligence, Figure 3 was presented incorrectly in the original publication. The corrected Figure 3 appears below. The authors state that the scientific conclusions are unaffected. This correction was approved by the Academic Editor. The original publication has also been updated.

## Figures and Tables

**Figure 3:**
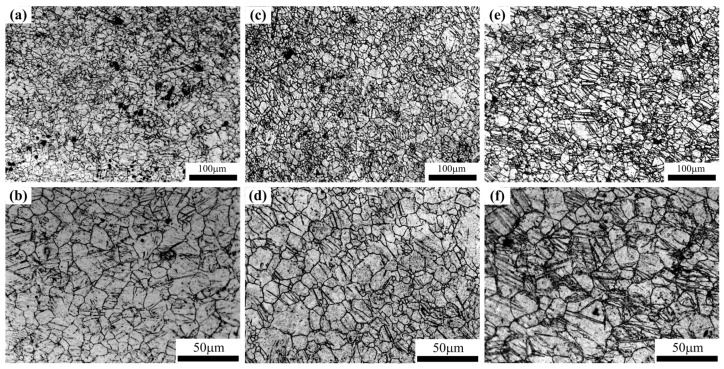
Microstructures of the ECAR-CB AZ31 Mg alloy samples prepared at different final rolling temperatures: (**a**,**b**) 350 °C, (**c**,**d**) 450 °C and (**e**,**f**) 550 °C.
